# Spanish L2 Chinese Learners’ Awareness of Morpho-Syntactic Structures in the Reading Comprehension of Splittable Compounds

**DOI:** 10.3389/fpsyg.2021.783869

**Published:** 2022-01-07

**Authors:** Ziming Lu, Ying Dai, Yicheng Wu

**Affiliations:** Department of Linguistics, Zhejiang University, Hangzhou, China

**Keywords:** splittable compounds, word recognition, linguistic comprehension, Verb-Object structure, decoding, L2 Chinese reading comprehension

## Abstract

Reading comprehension is never considered a simple task in linguists’ views as it requires a full set of linguistic knowledge, such as word decoding, understanding syntactic and morphological structures, and deriving proper meanings from these structures in a given context. Bearing the simple view of reading, the primary goal of this study is to explore whether the split presentation of Chinese splittable compounds influences the recognition of the compounds in second language (L2) Chinese reading comprehension, and how the reading skills, i.e., word decoding and linguistic comprehension, cooperate to complete this reading comprehension task. Splittable compounds (SCs) in Chinese are typically verbs composed of two constituents with limited separability. The separable property of SCs and their vague morpho-syntactic status are supposed to cause difficulties for L2 Chinese learners in recognizing the compounds. Especially for those whose native language manifests lexical integrity, the split presentation of the compounds may invite the L2 Chinese readers to process them with a mechanism different from that for their non-split forms. To the best of our knowledge, the efforts on investigating this issue are insufficient. In this study, 27 Spanish speaking L2 Chinese learners were invited to complete tasks including reading and interpreting 6 selected SCs in the split and non-split forms, rating their familiarities with each SC and reporting the syntactic category of the SCs based on their existing linguistic knowledge. The results, showed that the split presentation of SCs did cause challenges for L2 Chinese learners in recognizing the compounds in the reading process, regardless of their Chinese proficiencies. The L2 Chinese participants performed significantly worse in recognizing split SCs in salient Verb-Object structures than recognizing those in unsalient Verb-Object structures. These findings underscore the importance of linguistic comprehension in L2 Chinese in-text word reading comprehension and suggest words as the basic processing units.

## Introduction: Decoding and Linguistic Comprehension

Reading, though consisting of multiple “intricate workings of the human mind” (Huey, 1968: 6), can be viewed as simply involving two main tasks, decoding and linguistic comprehension (e.g., Fries, 1963; Venezky and Calfee, 1970; Perfetti, 1977; Gough and Tunmer, 1986; Hoover and Gough, 1990; Tunmer and Hoover, 1993). Decoding allows language in written form to be recognized, and linguistic comprehension includes the interpretation of given lexical information as well as syntactic structures and contextual implications. This simple view, as Tunmer and Hoover (1993) have emphasized, does not deny the complexities of the reading process, but addresses the importance of skills needed for language comprehension in addition to word decoding, such as determining the meanings of words in syntactic structures and deriving proper meanings from the structures in a given context.

Decoding in this simple view refers to a matching process from printed graphic representations to the mental lexicon. Considering orthographies across languages, two general types of decoding mechanisms have been proposed. The first, which is referred to as *phonological decoding*, hypothesizes that word recognition is accomplished by converting sequences of letters to phonological representations which are used to identify meanings in the mental lexicon (e.g., Tunmer and Hoover, 1993). The second mechanism proposes that graphic representations of words can be directly mapped onto the entries in the mental lexicon without phonological mediation. This strategy is referred to as *direct access* (e.g., Smith, 1973; Baron and Strawson, 1976). Phonological decoding is argued to be the primary route for reading alphabetic languages, especially for beginning readers, but when the orthographic representations have been established and enhanced in readers’ mental lexicon, words of high frequency can be recognized via direct access (e.g., Share, 1995; Schmalz et al., 2013). In other words, it is possible for alphabetic language readers, especially the advanced readers, to retrieve words automatically via the lexical cognitive route (direct access) (e.g., Coltheart et al., 2001; Häikiö et al., 2011). However, as Häikiö et al. (2011) addressed, the development of whole-word representations takes time and depends on the repetitive exposure to the words. Therefore, the phonological decoding strategy is still overprivileged and relied on when processing unfamiliar words in print.

For logographic languages, whose scripts do not specify phonological details, direct access is no doubt the dominant strategy for word recognition. Albeit a typical logographic script, the Chinese writing system contains a large portion of characters composed of both phonetic and semantic radicals, which also hypothetically allows both decoding strategies. Various studies have been conducted to prove the dual-route reading process for Chinese characters, but only weak sublexical phonological processing was found (e.g., Perfetti and Tan, 1998; Zhou and Marslen-Wilson, 2000; Zhang et al., 2012). There is no doubt that Chinese characters in print are able to activate phonological information, but it is still not clear if the activation is before or after the lexical access (Perfetti et al., 1992; Perfetti and Liu, 2006; Zhang et al., 2012). As Perfetti and Liu (2006) warned, phonetic radicals to Chinese characters are not the same as letters to words in alphabetic languages. Radicals themselves may be used as characters or words, while letters cannot. For example, 包 that can stand alone as a character/word also has a function of phonetic radical in words such as 跑 and 泡. However, 跑, 泡 and 包 are different in either/both onsets or tones in terms of pronunciation. In other words, phonetic radicals yield phonological information only partially related to the pronunciation of the character, and therefore it is unlikely that the pronunciation of unfamiliar characters can be precisely predicted via phonetic radicals. In contrast to alphabetic languages, the phonologically mediated semantic access is not reliable for orthographic processing in Chinese (e.g., Zhu, 1987; Feldman and Siok, 1999; Williams, 2013). By observing neural activities in visual word recognition, Zhang et al. (2012) found that although both phonological and semantic pathways were activated, the direct print-to-meaning mapping played a significant role in Chinese word reading comprehension.

According to Akamatsu (2005), the effects of first language (L1) writing system on second language (L2) reading cannot be easily reduced. Thus, given the differences between Chinese and alphabetic languages, the challenges faced by second language (L2) Chinese learners in Chinese character decoding is predictable. For Chinese learners whose native language is alphabetic, many of their script-dependent reading skills, such as the ability to distinguish shapes and sounds and to correspond sublexical written symbols to phonemes are not of much help in Chinese reading comprehension. Williams (2013) discovered that as phonetic radicals may not be reliable for predicting the whole character’s pronunciation, L2 Chinese learners’ phonological pathway for Chinese character decoding is less well established than the direct semantic pathway. Although L2 Chinese readers still tend to use phonetic radicals as decoding cues, as Williams (2013) argued, they use the radicals for orthographic disambiguation instead of phonological information.

A volume of literature has shown that the alphabetic language reading can be influenced by the orthography of words, such as orthographic shapes and letter case alternation (e.g., Fisher, 1975; Coltheart et al., 2001; Akamatsu, 2005). Although the direct lexical route is hindered, readers of alphabetic scripts can still achieve successful reading via the phonological route. In Chinese reading comprehension, since the phonological information is not as a strong predictor for lexical semantics as that in alphabetic languages, the presentations of words/characters are theoretically of great significance for accurate word/character decoding. The awareness of orthographic structures of characters has been argued of great importance to character decoding, especially for beginning Chinese readers (e.g., Tong et al., 2009; Yeung et al., 2013), but the orthographic skills in terms of radicals and sub-lexical processing are less strongly linked to multicharacter word reading (Pan et al., 2021). Successful character decoding cannot guarantee accurate Chinese reading comprehension, and it is necessary to address the difference between word recognition and character decoding in text-level reading. In Chinese, characters are the basic writing units, which can either stand alone as individual words or join to form multicharacter words. Thus, character decoding only partially contributes to multicharacter word reading comprehension. On one hand, words’ meanings are not always the simple composition of the semantics of each composing character, and on the other hand, in text-level reading comprehension, the formal continuity of a word’s composing characters in a string of characters should also be considered. In print, different from alphabetic scripts, Chinese words are not spaced, which implies that in text-level reading comprehension, sufficient linguistic analysis is required to determine which characters should be grouped for the retrieval of gestalt lexical semantics in the mental lexicon. The required linguistic analysis or comprehension includes recognition and use of grammatical structures (e.g., Gombert, 1992; Chik et al., 2012), and the structure awareness has been identified as a strong predictor in early Chinese reading comprehension (e.g., So and Siegel, 1997; Chik et al., 2012). Yeung et al. (2016) also addressed the interdependent relation between linguistic comprehension and word recognition in text-level reading comprehension. Pan et al. (2021) and McBride et al. (2003) demonstrated that character decoding and multicharacter word reading comprehension are associated but morphological awareness uniquely contributes to the latter. Thus, the accomplishment of Chinese multicharacter word reading comprehension requires not only accurate decoding of characters but also adequate analysis of both morphological and syntactic structures.

Within the simple view of reading, Chinese multicharacter word reading comprehension is in essence decoding as well, in terms of matching graphic representations with entries in the mental lexicon, though contextual information may be considered. Different from character decoding, however, multicharacter word recognition in Chinese is not independent of linguistic comprehension and especially in text-level reading, multicharacter word identification can hardly be completed without knowledge of lexical and syntactic structures. McBride et al. (2021) also suggested that in Chinese, the compounding feature and boundaries of a word in print also potentially complicate the reading process. Similar reading processes are hypothetically not common in alphabetic languages where the boundaries between words are clearly set by spaces in print. Thus, for L2 Chinese learners from the alphabetic language background, the complicated reading process composed of decoding and linguistic comprehension is supposed to be challenging. To the best of our knowledge, there is no previous research on L2 Chinese word reading comprehension considering the interdependent association between character decoding and linguistic comprehension.

The primary goal of this study, therefore, is to explore whether presentations of Chinese compounding words in print have impacts on L2 readers (without known reading disabilities in both L1 and L2) and how decoding and linguistic comprehension skills cooperate on the recognition of compounds in alternative presentations of compounds. Chinese splittable compounds (SCs) were selected as the research subjects. On one hand, the access to their gestalt lexical semantics requires adequate linguistic comprehension skills such as compound awareness, and on the other hand, the limited separability of composed morphemes provides possibilities of alternating their presentations without significant changes in their lexical meanings. The split forms of SCs further blur the boundaries of the words. Successful recognition of SCs in split forms theoretically requires cooperation of decoding and linguistic comprehension. Details about Chinese script reading and SCs are introduced next.

## Complexities in Chinese Reading Comprehension

In general, Chinese word reading comprehension requires information retrievals for both lexical and sublexical components (e.g., Koda, 2000), and thus studies on this topic usually focus on three aspects, viz., character recognition, compound awareness and word segmentation, as well as comparisons in these three aspects between Chinese and alphabetic orthographies, typically but not exclusively, English. As character recognition is mainly a matter of sublexical analysis that draws on knowledge of radical composition (e.g., Li and McBride-Chang, 2013), the latter two aspects are more relevant to the current study. Compound awareness and word segmentation in Chinese reading are somewhat interrelated. Compound awareness as a subset of morphological awareness can be defined according to Carlisle (2000) as the ability to reflect on and manipulate compounding components, i.e., individual characters in this research, while word segmentation concerns word recognition by identifying individual words embedded in unspaced character strings. During in-text reading, the former can be conceptualized as an operation of assembling and the latter as a dissembling process.

Given the visually unmarked word boundaries in written Chinese, word segmentation is no doubt important for fluent Chinese reading comprehension. It is by essence a chunking process, but different from spaced language scripts in which chucking occurs mainly at the phrasal level, it performs at the word level in Chinese (e.g., Valle et al., 2013; Yang, 2021). Li and Pollatsek (2020) postulated a model of word segmentation mechanism in Chinese reading comprehension. In this model, all characters in the perceptual span are supposed to be processed at the same time and multiple possible words containing these characters are simultaneously activated. According to this model, these possible words compete at levels of activation determined by frequency, compatibility with the context and other perspectives. When the contextually compatible words are recognized, the string segmentation is completed. This process is assumed to be particularly difficult for L2 Chinese learners whose L1 is spaced, as character decoding is not sufficient for accurate word recognition. It also requires linguistic comprehension such as compound awareness and contextual comprehension (e.g., Yen et al., 2012; Zang et al., 2016). The obscured definition of words in Chinese also incurs segmentation ambiguity with which L2 Chinese learners struggle a lot (e.g., Yang and Jiang, 2012). Yang (2021) reported that L2 Chinese learners felt stressed with word segmentation when reading Chinese aloud. Despite challenges as such, research shows that L2 Chinese learners are capable of applying morphological and syntactic knowledge, contextual information and prosodic implications to conduct word segmentation with high accuracy (e.g., Shen and Jiang, 2013; Yang, 2021).

Compound awareness is particularly a demanding skill for Chinese reading comprehension, for compounding is widely acknowledged as the primary word formation in Chinese (e.g., Taylor and Taylor, 1995; Huang, 1997; Packard, 2000; Ceccagno and Basciano, 2007; Chen et al., 2009; Tong et al., 2018). More than 75% of Chinese vocabulary are created via compounding (Taylor and Taylor, 1995). Anderson and Li (2005) hypothesized that Chinese reading requires higher compound awareness than English reading. The cross-language study conducted by McBride-Chang et al. (2005) has proven that compared to phonological awareness, compound awareness is more important in reading Chinese. Compound awareness is also suggested to contribute to lexical inference for L2 learners with Chinese words of different levels of semantic transparency (Chen, 2019). Along with it, cross-language transfer of compound awareness between the alphabetic script and the logographic script is also discussed in a volume of research (e.g., Kuo and Anderson, 2006; Zhang et al., 2010; Ramirez et al., 2011; Lin et al., 2018). Cross-language transfer, as defined by Genesee et al. (2006), is the tendency of learners to use linguistic knowledge of one language in learning another language. Ramirez et al. (2011) compared the performance of English language learners from Chinese and Spanish backgrounds in various morphological tests and found that Chinese English learners performed similarly to English monolinguals on English compound awareness while Spanish English learners, whose native language is the least productive with compounding, performed significantly lower.

In addition to the aspects presented above, another factor concerning word presentations, the linear organization of characters, in Chinese reading comprehension is also discussed in recent research. It has been proposed that words may not be processed in sequential order (e.g., Snell and Grainger, 2017; Mirault et al., 2018; Liu et al., 2021). Previous studies (e.g., Taft et al., 1999; Zhang et al., 2004; Bai et al., 2011) on this topic focus on reversible Chinese compound reading, i.e., transposed-character effect. This effect concerns the compounds which consist of the same morphemes but are presented in a reversed order. For example, the compounds 牛奶 “cow milk” and 奶牛 “milk cow” form a transposed-character pair. Liu et al. (2014) argued that as Chinese readers process compounds at the word level, the positional information at the character level is not that important. They also suggest that Chinese readers are not very sensitive to the position of composing characters in compounds. Beyond the word level, the alternation of word orders in Chinese signifies changes not only with meanings but also with the syntactic relation (e.g., Li and Thompson, 1981; Yang et al., 2009; Liu et al., 2021) and the transposed effect is not expected to be obvious, as readers may attach more attention to the word order. Liu et al. (2021) studied this transposed effect at the phrasal level (e.g., 她在看见山上云) and found that the transposed-word effect was influenced by context. The transposed-word effect was weakened if no context was provided before the transposed words. This observation is in line with the parallel processing model introduced above, and the readers can swap the implausible order of the words identified in the visual span based on the context. However, as Liu et al. (2021) pointed, it is not clear if such an effect exists in the L2 reading processing.

Inasmuch as words and phrases in Chinese are not clearly distinguished, it is potentially possible to explore the influence of another type of character organization on reading comprehension, namely the discontinuous presentation of words in print. According to the existing studies, morphological analysis is primarily applied to process the adjacent presentation of composing characters in word reading comprehension (Liu et al., 2014) and syntactic structure awareness is a demanding ability in reading the reversed order presentation of strings of words (e.g., Liu et al., 2021). It is not yet clear that when processing the discontinuous presentation of compounds that potentially straddles lexical and phrasal structures, which linguistic knowledge is applied and how the choice impacts the reading comprehension. The L2 reading of Chinese SCs in their split forms can be taken as a case in point.

## Chinese SCs and L2 Chinese Learners’ Acquisition

Chinese SCs, which are known as “*lihe ci”* among Chinese linguists, like most Chinese words, are typically composed of two characters (or morphemes), and each character can stand alone as an individual word. They are commonly used as verbal structures in modern Chinese (Siewierska et al., 2010; Wang, 2011; Xu and Li, 2014). According to the syntactic relation between the two composing characters, SCs can be roughly grouped into three subtypes, verb-object SCs (V-O SCs), verb-complement (resultative or directional) SCs (V-C SCs) and subject-verb SCs (S-V SCs), among which V-O SCs are the most productive (Zhu, 2006; Wang, 2011). Zhu (2006) also identified a coordinative subtype, such as 游泳 “swim,” in which the two morphemes are synonymous, but many linguists (e.g., Wang, 2011; Xu and Li, 2014) treat them as V-O structures as well. Thus, they are also grouped into the V-O subtype but labelled as pseudo V-O SCs in the current study.

What makes this type of compound special is the fact that the two composing characters can be separated by inserting limited types of functional or grammatical structures. In their respective corpus-based studies, Siewierska et al. (2010) and Wang (2011) identified several types of constituents that can be inserted between the two characters of SCs, among which aspect markers, such as 了 *le* (perfective), 过 *guo* (experiential), 着 *zhe* (durative), are the most common (more than 50%). Quantificational expressions, classifier structures, and pre-modifiers that modify the tails of SCs are all ranking high on the list of typical insertions in split SCs. The combinations of these constituents are illustrated in (1).

(1) a. Aspect marker

说过话 speak ASP speak “to have talked”

b. Quantificational expression

读点书 read some book “to do some reading”

游两次泳 swim two times swim “to swim twice”

c. Classifier structure

吃三顿饭 eat three CL rice “to have three meals”

跑个步 run CL step “to have a run”

d. Pre-modifier

排长队 queue long row “to queue in a long line”

跳5分钟的舞 dance 5 min dance ‘to dance for 5 min’

Despite the split usages of SCs, their lexical semantics is not necessarily influenced. As indicated in the examples above, the meanings of the split forms are not simply the combination of individual characters, and the idiomatization between the two composing characters of SCs cannot be neglected.

Thus, the dispute about SCs in literature has centered on their morpho-syntactic status, as no one can turn a blind eye to the conflicts between the gestalt lexical semantics of SCs and their violation of the Principle of Lexical Integrity (PLI, Lapointe, 1980), which states morphological words cannot be separated by syntactic operations. Chao (1968) advanced an “ionization” view of SCs by an analogy with chemistry in the sense that a compound is a unit whereas its components can separate. Huang (1984) rejected Chao’s idea of “ionizable words,” as this concept is hard to be defined precisely. Instead, following formal principles, Huang (1984) grouped SCs as phrases but with idiomatic meanings. Lü (1979) provided a prudential solution, suggesting the existence of a transitional category in morpho-syntax, and it is widely accepted. Unlike most verbal phrases, Li and Thompson (1981) pointed out that the idiomaticity and separability of SCs are not predictable. Some SCs (such as 吃饭 “lit. eat rice” = = “eat”) are semantically transparent but some are not (e.g., 担心 “lit. carry heart” = “worry”). Although, as Wang (2011) observed, for most SCs, non-split forms are their normal forms, the separability of individual SCs is not the same. For example, 帮忙 “help,” is more frequently used in the split forms compared with走私 “smuggle” that is rarely split. Hence, SCs form a continuum that represents a transition from phrases to compounds. Siewierska et al. (2010) also approved this view and further specify that SCs split by aspect markers are compounds, but those split by modifiers (including nominal classifiers and other pre-modifiers) attached to the tails are phrases.

The morphological and syntactic complexities of SCs also create great challenges for L2 Chinese learners, and many studies have been conducted on this topic. All existing studies notice that L2 Chinese learners are reluctant to use SCs in their split forms when needed, regardless of their language backgrounds or their Chinese proficiency levels (e.g., Ma, 2008; He, 2009; Shen, 2019). The learners tend to find the equivalence of SCs in their L1 and observe their native lexical rules when using SCs. For example, VO SCs are generally intransitive in Chinese, but they have transitive verbal equivalence in other languages. A typical form of errors made by Korean L2 Chinese learners, as observed in Lin (2005) and Yang (2006), was that they often added the objects directly after the SCs following the correct word order in Korean. This error type was also identified among learners from English and Russian backgrounds (Qin, 2020; Valeriya, 2021). Hou (2021) made some interesting observations by analyzing the errors involving SCs made by Japanese speaking learners. Since Chinese characters, namely Kanji, form a portion of the Japanese vocabulary, many Chinese SCs have homographs in Japanese, albeit syntactically and semantically different. Some Japanese homographs are words that cannot be split in the same way as their counterparts in Chinese, but some are phrases. Japanese speaking learners typically avoided using split forms of Chinese SCs whose Japanese homographs are lexical. When using Chinese V-O SCs whose Japanese homographs are phrasal, Japanese speaking learners were found to reverse the order of the two morphemes in split forms. Learners from English (Qin, 2020), Spanish (Xing, 2021) and Russian (Valeriya, 2021) backgrounds prefer to place aspect markers after SCs instead of inserting them in between. The transfer of morphological knowledge in L1 may be the cause for these errors, as English, Spanish and Russian, which are typologically different from Chinese, rely on inflectional changes at the end of verbs to express aspectual meanings.

All these existing studies imply that the main cause for the errors made by L2 Chinese learners when using SCs is the learners’ low awareness of this structure. The learners fail to identify the compounds which are splittable in Chinese and therefore carry over the parameter settings in their native languages to Chinese. As Schwartz and Sprouse (1996) hypothesized, this transfer is typically observed at the early stage of second language acquisition and may be weakened along with the development of second language proficiency. Shen (2019) conducted a study on the familiarity awareness and comprehension of SCs among L2 Chinese learners in the United States. The results show that there was a significant increase of mastery of split SCs between the end of the second-year and the third-year study, yet the overall awareness of the split use among all proficiency levels remains low.

To the best of our knowledge, however, there lacks such research exploring the comprehension or recognition of split SCs in the L2 reading process. Challenges faced by L2 Chinese readers lying in decoding and linguistic comprehension can be reasonably assumed. In split forms, the composing characters of SCs are not presented adjacently in a string of characters, and the segmentation process, therefore, is conducted beyond the word level. The L2 reading process of split SCs is therefore complex. It is possible for L2 Chinese learners to map the two discontinuous characters onto separate entries in their mental lexicon and fail to extract their gestalt lexical semantics. It is also possible for L2 readers to access a neighbor entry that is similar in form to the target word based on the incomplete presentation of the SC. In terms of linguistic comprehension, the separability of SCs straddles the lexicon-syntax division and runs afoul of PLI, which is rare in other languages. L2 Chinese learners, especially those from alphabetic backgrounds, lack experience in processing such structures in their L1. They may still rely on their native linguistic knowledge in reading SCs, which may prevent them from appropriate SC structure analysis.

The current study is designed to validate the hypothesis that both compound awareness and syntactic structure awareness are activated in the Spanish L2 reading comprehension of the split SCs. The selection of Spanish L1 was based on the following facts. First, it does not allow sublexical elements of compounds to be moved by syntactic rules following the PLI (e.g., Slobin, 1996; Los et al., 2012; Iacobini, 2015). Second, it differs dramatically from Chinese in both script types and primary word-formation processes. Spanish is an alphabetic language in which letter-phoneme correspondence is highly predictable, and therefore, the phonological decoding is privileged for Spanish speakers. In contrast, Chinese is a logographic language, and its characters as the basic writing units do not correspond to sounds in the same way. As argued above, in Chinese reading comprehension, the association of phonology and character/word decoding is not as strong as that in Spanish. In addition, due to the lack of derivational morphemes, compounding is widely acknowledged as the dominant word formation in Chinese, while it is the least productive in Spanish. As Ramirez et al. (2011) demonstrated, compound awareness among Spanish speakers is very low. These contrasting features may potentially cause challenges for Spanish L2 Chinese learners in decoding and linguistic comprehension when encountering split SCs in Chinese reading comprehension.

Accordingly, we designed a reading-interpreting online test for Spanish L2 Chinese learners. The word reading comprehension was assessed by their recognition accuracy reflected on the split SC interpretations, and the skills related to linguistic comprehension applied were discussed by analyzing their reading strategies. The translations of isolated non-split SCs were taken as a reference of the participants’ decoding skills. The role played by their L1 linguistic knowledge in reading split SCs was also investigated. Thus, the current study aims to address the following three specific research questions:

1. To what extent are the Spanish L2 Chinese learners able to recognize SCs in their split forms?
2. What are the challenges faced by the Spanish L2 Chinese learners in the in-text split SCs reading comprehension? What are their strategies to tackle the challenges?
3. To what extent are the Spanish L2 Chinese learners sensitive to the structures that deviate from PLI held in their L1 linguistic knowledge?

## Materials and Methods

### Participants

A total of 27 L2 Chinese learners from Spanish background participated in the study. None of them was known for nonverbal reasoning or other learning disabilities. In order to minimize the influence of other languages, such as Japanese and Korean which orthographically resemble Chinese, all the participants selected do not have or had only limited knowledge of other East Asian languages. In addition, the family languages used by the participants did not include Chinese. Based on the information collected from a family questionnaire designed by the researchers, all the participants had received formal and intensive Chinese language instructions for at least one year and were able to recognize daily used Chinese characters in print. Among them, 24 have passed the HSK test (a standardized test for Chinese proficiency) of different levels (2 of Level II, 10 of level III, 9 of Level IV, and 6 of Level V), and 3 have achieved the advanced Chinese proficiency equal to HSK IV and above as assessed by Zhejiang University. To create a control group, 35 Chinese native speakers from universities in Hangzhou (China) who were not linguistic majors were invited to participate in the study. They were assumed highly literate and able to recognize the non-split SCs selected in the test.

### Design and Materials

6 SCs of two internal structures (3 of pseudo V-O structures and 3 of V-O structures) were selected and presented in their split and non-split forms (see Table 1). There were altogether 14 sentences hosting the split forms of the 6 SCs, and the sentence codes were given following the order of presentation in this reading test.

**TABLE 1:** Testing materials.

SC Types	Non-split SCs	Sentence Codes	Split SCs embedded in sentences
Pseudo V-O SCs	跳舞	S4	今天他还会跳两支舞。
			Today, he will still dance two dances.
		S9	在 party 上，大家都跳了舞。
			At the party, everyone danced.
	游泳	S5	他只能游三十米的泳。
			He can only swim 30 meters.
		S10	他只能游三十米的泳。
			He swims twice ever week.
		S13	今天我们先出去游个泳吧。
			Let’s go swimming first today.
	学习	S6	明天考试，今天我要学点习。
			There is an exam tomorrow, so I need to study a bit.
		S11	他过去两年都没有学过习。
			He hasn’t been studying for the past two years.
V-O SCs	吃饭	S2	我要吃好多好多饭。
			I want to eat lots and lots of food.
		S12	他现在正吃着饭。
			He is eating right now.
	跑步	S1	公交车来了，他快速跑了几步。
			The bus was coming, and he quickly ran a few steps.
		S7	他每天晚上跑三十分钟步。
			He jogs 30 min every evening.
		S14	他早上想去海边跑个步。
			He wants to jog at the sea.
	排队	S3	学生在食堂排了很长的队。
			The students formed a long queue in the food hall.
		S8	他排了一晚上的队，所以很累。
			He queued the whole night so was very tired.

Considering the participants’ Chinese proficiency levels, all the characters used to compose the materials were selected based on the HSK Level III vocabulary list (Hanban, 2012), which ensured most of these words and characters involved in the test were familiar to most of the participants. Productivity and semantic transparency were also considered during the process of SC selection, as these factors are supposed to have an impact on compound awareness in literature (Zhang et al., 2010; Chen, 2019). The SCs selected for the test were regarded as productive mainly for the following two reasons. First, Wang (2011) has discovered that most SCs (77%) designate activities commonly experienced or conducted by people. Second, the 6 SCs were all picked from the HKS vocabulary list for learners who are expected to master about 2,000 Chinese words. Thus, it is reasonable to assume they are frequently used in daily life and therefore productive.

**FIGURE 1:**
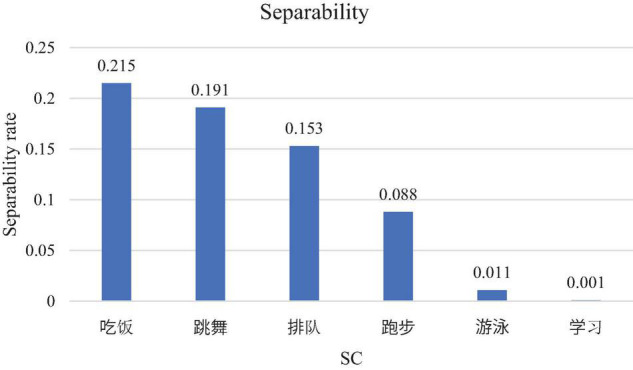
Separability of the selected SCs.

Although inevitably, the selected 6 SCs varied in semantic transparency and separability, we tried to control these impacts. Pseudo V-O SCs, in general, are less transparent than V-O SCs (e.g., Wang, 2011) because their composing morphemes by nature do not have the V-O relation. We then selected the pseudo V-O SCs (跳舞 “dance,” 学习 “study,” 游泳 “swim”) that are originally V-V structures composed by two synonyms but treated like V-O structures in modern Mandarin. The three V-O SCs (吃饭 “eat,” 跑步 “run,” 排队 “queue”) are all semantically transparent, and each composing character can correspond to a single word in Spanish. With the BCC corpus developed by Beijing Language and Culture University Corpus Centre, the separability of each selected SC was calculated based on the formula proposed by Wang (2011), as shown in Figure 1.

In order to reflect the real use of split forms of SCs while minimizing the influence of context, simple sentences hosting the split SCs were selected from the BCC corpus. The choice of insertions, which may influence the readers’ syntactic analysis, was also carefully considered. Table 2 lists the types of insertions and their corresponding sentence codes.

**TABLE 2:** Insertions.

No.	Insertion types	Sentence codes		
1	aspect marker	S9	S11	S12
2	verbal classifier	S10		
3	nominal classifier	S4		
4	*ge*	S13	S14	
5	quantificational expression (Quan(titative))	S2	S6	
6	quantificational expression (Temp(oral))	S7		
7	*de* structure modifier (Qual(itative))	S3	S5	
8	*de* structure modifier (Temp(oral))	S8		
9	fake structure	S1		

In 3 testing sentences (S9, S11, and S12), only aspect markers 着 *zhe* (progressive), 过 *guo* (experiential), 了 *le* (perfective) were applied. The nominal classifier and the verbal classifier with numerals were chosen for 2 testing sentences (S4 and S10) forming a contrast, as the former is typically used to quantify entities denoted by the adjacent nominals, and the latter indicates the occurrence frequency of the activities designated by verbal constituents. 个 *ge* was listed as a separate type of insertion (in S13 and S14) from other nominal classifiers because个 *ge* in the postverbal position has been argued for aspectual function in the relevant literature (e.g., Shi and Lei, 2004; Wu, 2004). Quantitative expressions denoting quantity (S2 and S6) and temporal duration (S7) as well as premodifiers in the form of 的 *de* structure denoting quality (e.g., 很长的 “very long” in S3 and 三十米的 “of three meters” in S5) and temporal duration (such as 一晚上的 “a night of” in S8) were all used as insertions. For the sake of grammaticality and acceptability, aspect marker 了 *le* was inserted together with other types of insertions in the testing sentences S1, S3, and S8. The length of the insertions ranged from one to four characters, and the average was two.

Xu and Li (2014) have warned that the translations of split SCs are less stable than their non-split forms and thus we tried to limit the diversity of possible Spanish correspondences across individuals by manipulating the structure of the sentences. In addition, we also composed a “fake split form” involving the characters of 跑步, as in 跑了几步 “ran a few steps.” It is a phrasal structure, whose syntactic structure and meaning were both different from the split form of the SC. In this “fake structure,” the verb 跑 “run” is sufficient to designate the activity, and the following noun phrase (NP) is its object. In the NP, the noun modified by the quantifier is referential and can be identified independently from the activity. We expected participants’ diverse performances regarding these features.

### Procedure

Due to the COVID-19 pandemic, each participant was accessed online via Zoom or DingTalk, in a 30-40 min’ session. The whole session consisted of two parts. The first part involved 14 reading-interpreting tasks, and the second was a brief interview regarding the SCs chosen in the test. After being explained the instruction, the participants were first required to interpret each sentence involving the split SCs as presented one by one on the screen into Spanish. Then, they needed to type and send the final interpretation they just said via message. We assumed that a very recent previous reading experience of non-split SCs might influence the recognition of their split forms, and to avoid such possibility, each participant was first expected to interpret 14 sentences. They were allowed to skip sentences if they could not complete the task. In the second part, 6 non-split SCs involved in Part One sentences were presented without any context, and the participants were asked to translate them into Spanish. In addition, the participants needed to rate how familiar they were with these 6 SCs ranging from 0 to 5. “5” meant that they were very familiar with the SC, while “0” indicated that they rarely came across with the expression. After this, the participants were asked to identify the grammatical category of each SC as presented in their non-split forms. No clear definitions of words and phrases were given, and the participants were expected to make the decision based on their own linguistic knowledge and intuition. For the convenience of statistical analysis, the phrasal status of SCs was coded as “1” and the lexical status was coded as “0.” Finally, they were interviewed about their previous Chinese learning experience related to SCs. During the whole session, participants were free to communicate with the examiners, comment on the tasks and express their struggles.

In addition, the control group were invited to complete an online questionnaire, in which the 14 testing sentences were listed, and all the 6 SCs embedded in these sentences were presented following each sentence. The control group needed to select the correct SC involved in each sentence. If they did not select the correct SC or stated none of the given SCs appeared in the sentence, they were counted as unsuccessful cases in the task.

### Data Analysis

By message documentation and video transcription, the original data were collected. The data were further coded by the two raters, judging whether the participants could recognize each SC in their split and non-split forms. The internal consistency reliability was 99%. The successful recognition of an SC was marked when the SC was interpreted into Spanish correctly, and the results were measured by nominal variables with two labels: Recognized (R) and Not Recognized (NR). IBM SPSS 26.0 and Microsoft Excel were used to conduct the statistical analyses.

First, we compared the reading comprehension performance of split SCs in 14 sentences between participants of different Chinese proficiency levels using related-samples non-parametric tests, i.e., Wilcoxon test. Second, we created a table to show the recognition situation of each non-split SCs among the Spanish participants. We considered that failure in recognizing the non-split SCs indicated the lack of knowledge of the words, and it may interfere with the results for the central interest. Thus, the rest report on the recognition of each split SC involved only the performances of the participants who successfully recognized the word’s non-split form. We presented the results of the split SCs’ recognition task based on the table created in the second step and summarized the accuracy rate among Spanish participants. After this, we compared and contrasted the average error rate in recognizing the two types of SCs: pseudo V-O SCs and V-O SCs, aiming to discover the influence of SCs’ internal structure. Finally, the differences in error rates related to each insertion type (except “fake structure”) were examined by an independent-samples non-parametric test, namely, Mann-Whitney *U* test.

## Results

The responses of a total of 27 Spanish participants and 35 Chinese participants were included in the statistical analysis. According to their HSK results, all the Spanish participants were initially grouped into two: intermediate level (HSK levels II and III) and advanced level (HSK level IV and V). The descriptive data of the two groups concerning the split SC recognition were presented in Table 3 along with the control group. Three types of errors related to SC recognition were observed among the Spanish participants. One was that the participants failed to give an interpretation of the sentence due to the split structure, and the second was that they mistook the given split SC for another word and produced an incorrect interpretation. For example, one participant interpreted 跑步into *caminar* “walk” in all the sentences hosting the split 跑步 but successfully recognized it when presented in its non-split form. A third type was that some participants improperly interpreted the tail morpheme in the SC. For example, 游30米的泳was interpreted into *nadar 30 piletas* “to swim 30 swimming pools,” which does not make any sense. In the control group, no one selected the incorrect SCs but some stated none of the given SCs appeared in the sentences.

**TABLE 3:** Split SC recognition in each testing sentence.

Sentences	Intermediate (*N* = 12)			Advanced (*N* = 15)			control (*N* = 35)		
	R	NR	Error rate	R	NR	Error rate	R	NR	Error rate
S1	3	9	75.00%	5	10	66.67%	34	1	2.86%
S2	11	1	8.33%	14	1	6.67%	34	1	2.86%
S3	7	5	41.67%	14	1	6.67%	35	0	0.00%
S4	9	3	25.00%	13	2	13.33%	35	0	0.00%
S5	11	1	8.33%	14	1	6.67%	35	0	0.00%
S6	12	0	0.00%	15	0	0.00%	35	0	0.00%
S7	10	2	16.67%	13	2	13.33%	35	0	0.00%
S8	8	4	33.33%	13	2	13.33%	35	0	0.00%
S9	12	0	0.00%	15	0	0.00%	35	0	0.00%
S10	11	1	8.33%	14	1	6.67%	35	0	0.00%
S11	12	0	0.00%	15	0	0.00%	34	1	2.86%
S12	11	1	8.33%	13	2	13.33%	35	0	0.00%
S13	10	2	16.67%	13	2	13.33%	35	0	0.00%
S14	10	2	16.67%	9	6	40.00%	34	1	2.86%

*R stands for the count of recognized cases; NR is for the count of not recognized cases.*

In addition, the related-samples non-parametric tests, i.e., Wilcoxon tests were conducted to compare the error rate in recognition between the groups of different Chinese proficiency levels. There was not a significant difference in the performance between intermediate and advanced L2 learners (test statistic = 16, *p* = 0.147), but the control group performed significantly better than the advanced L2 Chinese learners in terms of recognizing the split SCs (test statistic = 0, *p* = 0.001). As the Spanish L2 learners’ Chinese proficiency was not the main contributor to the recognition of split SCs in the current study, it was not considered as a variable in the following report and analysis.

### Recognition of Non-split SCs

Table 4 presented the accuracy and error rates of recognizing the 6 non-split SCs without contexts. It was shown that all 27 participants correctly recognized the non-split 跳舞, 学习 and 吃饭. The preponderant percentage of successful recognition (100% for 跳舞, 学习 and吃饭, 96.30% for 游泳, 88.90% for 排队 and 81.50% for 跑步) and the general familiarity across participants with these SCs (Mean = 4.5, SD = 0.57) showed that the SCs selected were suitable for the participants’ Chinese proficiency levels on the whole. In the interview, all participants deemed the words basic and easy to recognize, although some of their translations were not correct.

**TABLE 4:** Recognition of non-split SCs.

SC Types	Non-split SCs		Count	N %
Pseudo V-O SCs	跳舞	R	27	100.00%
		NR	0	0.00%
	游泳	R	26	96.30%
		NR	1	3.70%
	学习	R	27	100.00%
		NR	0	0.00%
V-O SCs	吃饭	R	27	100.00%
		NR	0	0.00%
	跑步	R	22	81.50%
		NR	5	18.50%
	排队	R	24	88.90%
		NR	3	11.10%

*R stands for the count of recognized cases; NR is for the count of not recognized cases.*

### Recognition of Split SCs

Table 5 based on the results from Table 4 exhibited the recognition accuracy of SCs in their split forms among the participants. As shown in Table 5, all those who could recognize non-split 学习 succeeded in recognizing its split form in S6 and S11. For 跳舞, although some Spanish participants made errors with S4, all of them successfully recognized it in S9. For the V-O SC吃饭, a similar pattern was observed. 2 Spanish participants failed in recognizing 吃饭in S2, but no one had any problem with S12. As to the rest of the SCs, 游泳, 跑步 and 排队, wrong or null answers were given in each recognition task with their split forms, even though they were all correctly recognized in the non-split forms. Specifically, in recognizing 游泳, among all the participants who know the non-split SC, 2 failed in S5, 2 made a mistake in S13, and one did not successfully recognize it in both S10 and S13. In S3 and S8 that hosted 排队, three wrong or null responses were observed in each, and one participant failed in both. Most participants struggled with S1, which was a phrasal structure. The error rate of this fake split SC structure was as high as 68.20%. In contrast, only one Spanish participant made a mistake with 跑步 in S7, and another 4 could not recognize it in S14.

**TABLE 5:** Recognition of split SCs.

SC Types	Non-split SCs			Split SCs				
		Spanish			Spanish		Control	
		R	N%		R	N%	R	N%
Pseudo V-O SCs	跳舞	27	100.00%	S4	22	81.50%	35	100.00%
				S9	27	100.00%	35	100.00%
	游泳	26	96.30%	S5	25	96.20%	35	100.00%
				S10	25	96.20%	35	100.00%
				S13	23	88.50%	35	100.00%
	学习	27	100.00%	S6	27	100.00%	35	100.00%
				S11	27	100.00%	34	97.14%
V-O SCs	吃饭	27	100.00%	S2	25	92.60%	34	97.14%
				S12	27	100.00%	40	100.00%
	跑步	22	81.50%	S1	7	31.80%	34	97.14%
				S7	21	95.50%	35	100.00%
				S14	18	81.80%	34	97.14%
	排队	24	88.90%	S3	21	87.50%	35	100.00%
				S8	21	87.50%	35	100.00%

*R stands for the count of recognized cases.*

In addition to Table 5, it was interesting to find that 2 participants who could not recognize 跑步 in its non-split form reported the correct interpretation when the composing characters of 跑步 were presented separately. According to the above observation, the participants did not show consistency in recognizing the same SC in different sentences, suggesting that the contribution of structures (both morphological and syntactic) should be taken into consideration. Thus, the following sections reported the influence of morphological structures and syntactic structures on the reading task.

### Pseudo or Non-pseudo V-O Contrast

Based on the recognition accuracy of split SCs among the Spanish participants, we observed that it seemed more challenging for the L2 Chinese learners with V-O SCs (吃饭, 跑步 and 排队) than the pseudo ones (跳舞, 游泳 and 学习). To further illustrate the difference, the average error rate of these two types of SCs was calculated. As shown in Table 6, the average error rate with V-O SCs (15.50%) was almost three times higher than that with pseudo V-O SCs (5.22%).

**TABLE 6:** Error rate concerning the SC types.

SC type	SCs	Sentence	Error	Sentence	Error	Sentence	Error	Average	Type
		codes	rate	codes	rate	codes	rate		Average
Pseudo V-O SCs	跳舞	**S4**	18.50%	**S9**	0.00%			9.25%	5.22%
	游泳	**S5**	3.85%	**S10**	3.85%	**S13**	11.54%	6.41%	
	学习	**S6**	0.00%	**S11**	0.00%			0.00%	
V-O SCs	吃饭	**S2**	7.41%	**S12**	0.00%			3.70%	15.50%
	跑步	**S1**	68.20%	**S7**	4.50%	**S14**	18.20%	30.30%	
	排队	**S3**	12.50%	**S8**	12.50%			12.50%	

**FIGURE 2:**
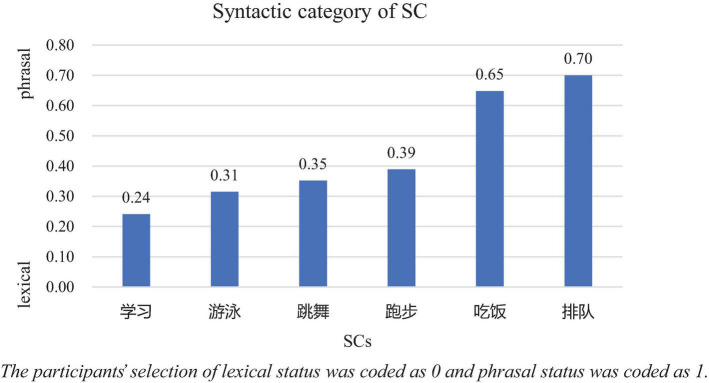
Syntactic category of SCs based on the L2 Chinese learners’ intuition.

Also, in the following interview, all participants reported that they were familiar with these SCs, but they were indecisive when identifying the SCs’ syntactic category. Albeit the struggles, a general agreement on the lexical status of pseudo V-O SCs (学习, 游泳and 跳舞) could be observed, as shown in Figure 2.

排队 as shown in Figure 2, was considered as a phrase by most of the Spanish participants, and the error rates with S3 and S8 were high as well. In a similar fashion, 吃饭is located toward the phrasal end in Figure 2 and the error rate with S2 hosting 吃饭was high, too. However, no one failed in recognizing 吃饭in S12, and the most obvious difference between the two sentences lies in the types of insertions between the two composing characters.

### Contribution of Insertions to the Recognition of Split SC

The observation in 3.3 regarding the types of insertions encouraged us to investigate the influence of this aspect on the recognition of split SCs. We created Table 7 presenting the average error rates of each insertion type in a low to high order. As the “fake structure” was deliberately composed as a contrast, it was applied in addition to the 8 types of insertions as a polar indicator.

**TABLE 7:** Error rate concerning the insertion types.

No.	Insertion type	Sentence codes	Error rate	Sentence codes	Error rate	Sentence codes	Error rate	Average
1	aspect marker	**S9**	0.00%	**S11**	0.00%	**S12**	0.00%	0.0%
2	quantificational expression (Quan)	**S2**	7.41%	**S6**	0.00%			3.7%
3	verbal classifier	**S10**	3.85%					3.8%
4	quantificational expression (Temp)	**S7**	4.50%					4.5%
5	*de* structure modifier (Qual)	**S3**	12.5%	**S5**	3.8%			8.2%
6	*de* structure modifier (Temp)	**S8**	12.5%					12.5%
7	*ge*	**S13**	11.5%	**S14**	18.20%			14.9%
8	nominal classifier	**S4**	18.50%					18.5%
9	fake structure	**S1**	68.20%					68.2%

It was shown that the participants found reading the “fake structure” challenging. Insertions of nominal classifiers (including *ge*) and premodifiers in the form of *de* structure also generated challenges for the participants in the reading comprehension. The results in Table 7 implied that Spanish participants’ reading process was less disturbed when reading sentences in which verbal classifiers and quantificational expressions (Quan and Temp) were used as insertions. Thus, Table 7 vaguely suggested a processing difficulty scale of insertion types, on which quantifiers (temporal and quantitative), verbal classifiers and aspect markers were located toward the easy end.

For a more general observation, the insertions were further merged into two groups based on their functions. Nominal classifiers (including *ge*) and *de* structures formed a group as they are typically used as premodifiers for nouns. Quantificational expressions that can be used as adjuncts were grouped together with aspect markers and verbal classifiers, as all of them function over verbs or verbal phrases. The former was referred to as modifier group and the latter was adverbial/aspectual group. The “fake structure” was not merged with either group. An independent-samples non-parametric test (Mann-Whitney *U* Test) was adopted to examine the group differences. The result indicated that the modifier group was significantly more challenging than the adverbial/aspectual group (test statistic = 3, *p* = 0.01).

## Discussion

To summarize the results, we found that the split presentations of SCs had an impact on Chinese word reading comprehension among the Spanish participants as they failed to recognize the known SCs embedded in some simple sentences. Their recognition performance was significantly worse than Chinese native readers, and the contribution of their Chinese proficiency was not statistically significant as shown in the result. The awareness of syntactic relation between two composing characters of SCs was observed as a significant contributor to split SC recognition. The participants performed significantly better with pseudo V-O SCs than with V-O SCs. Also, there was a general agreement among the Spanish participants on that compared to the SCs of the V-O internal structure, pseudo V-O SCs were more lexical. Taking the insertions into consideration, we found that the split SCs with insertions of pre-nominal modifiers (modifiers of *de* structures and nominal classifiers) were more challenging for the participants than those separated by adverbial and aspectual expressions (quantificational expressions, verbal classifiers and aspect markers).

To further elaborate on the first research question, the failure in recognizing the known SCs in split forms indicates that the word decoding process of Spanish L2 Chinese learners was interrupted by the discontinuous presentation of the composing characters. As described in 3.1, three types of errors were observed related to SC recognition, which can be referred to as null recognition, misrecognition and over-recognition. For null recognition, 7 participants were not able to retrieve the words in their mental lexicon based on the representations in print in 13 sentences. However, they could successfully map the non-split forms onto the correct lexical entries, which indicates that the separate presentation of SCs’ composing characters hindered the direct lexical access. In the cases of misrecognition, which was also the error type of the most occurrences as exhibited in the data (*N* = 24), the Spanish participants confidently matched the orthography with the incorrect semantics. For example, 10 participants interpreted the split forms of 跑步 “run” in 14 sentences as *caminar*, which is equivalent to 散步 or 走步 “walk” in Chinese. 4 participants mistook split 游泳 “swim” for 旅游 “travel,” as they used *viajar* in 5 sentences in their responses. However, none of these participants made such errors in the non-split SC translation task. Similar to the first type, the participants possibly started the decoding process based on the incomplete word form and mapped it onto a different meaning whose representation in orthography is similar to the target SC in print. Over-recognition was not as common as the other two, and only 4 people overtly interpreted 泳 in 游泳 as *pileta* “swimming pool” in 4 sentences. In other words, the participants attempted to decode the composing characters of an SC separately and retrieve each character as an independent word. This type is different from misrecognition in that in over-recognition the tail morpheme of the SC was salient for the reader. All these errors were related to the discontinuous presentations of SCs and the participants’ awareness of this morphological structure that was not strong enough to help them pick and join the composing characters together in the perceptual span.

With respect to the second research question, we attempted to address the challenges faced by L2 Chinese learners in reading split SCs and their strategies to tackle them. Taking the statistical analysis together, the main factors affecting L2 Chinese learners’ SC recognition were the composing characters’ syntactic relation and types of insertions. More precisely, measures indicated that the awareness of the V-O structure was disruptive to split SC reading. The participants’ performance was better with pseudo V-O SCs, in which the semantic relation between the two characters is obscure, than with V-O SCs, in which the semantic relation is transparent. Furthermore, in consistent with the statistical results, most misrecognition and null recognition occurred with SCs inserted by modifiers of *de* structures, nominal classifiers, which are typical constituents of NPs. Yang (2021) reported that L2 Chinese learners tended to attach classifiers and modifiers to the following characters when conducting word segmentation tasks. Thus, the tail character in an SC was likely to be grouped with these insertions as nominal, and with the preceding verbal morpheme, it was natural to establish a V-O relation between the two parts. On the contrary, the insertions that were shown easier to process in SCs, such as aspect markers, verbal classifiers and quantitative expressions are more verbal related and did not promote the saliency of object role of SC tails.

The difference in recognizing split SCs of the salient V-O structure and those of the unsalient V-O structure implied that it was unlikely that both types of SCs were processed via the same mechanism. The salient V-O structures were possibly processed as phrases, and the composing characters were decoded separately as single-character words. The unsalient ones were comprehended via mapping the composing characters jointly onto a single entry in the mental lexicon. In the interviews, when asked about the criteria applied in categorizing SCs, most participants expressed that based on their native linguistic knowledge, if the SC could be analyzed as a V-O structure, it was less likely to be a word. Thus, it is reasonable to predict that L2 Chinese learners may fail to recognize the split SCs of the salient V-O structure with the absence of compound awareness. In other words, they may fail to decode the SCs in these structures as a whole and instead, process the expressions character by character. This assumption was approved in many responses of the participants. When interpreting SCs inserted by modifiers of *de* structures and nominal classifiers, the participants tended to express the tails overtly. For example, many participants struggled with 跳两支舞 “dance two dances” in which a nominal classifier structure was inserted. They explained that it sounded strange in their native language if 舞 “dance” was expressed. The over-recognition errors were also related to this assumption. The participants who over-recognized 泳 as *pileta* explained that the meaning seemed incomplete if this piece of information was not expressed. Thus, we predicted that the salient object role of the character 泳 motivated decoding.

The split presentation of SCs was not unhelpful at all. We noticed that L2 Chinese learners relied on composing characters in processing less familiar SCs in some cases. For instance, 2 participants who could not recognize 跑步 in its non-split form reported correct interpretations when the composing characters of 跑步 were presented separately. They explained in the following interview that they inferred the meaning of the expression from the context and also by recognizing the character 步 that indicates either running or walking. This strategy is in line with the processing architecture proposed in previous research (Taft and Forster, 1976; Taft and Ardasinski, 2006; Treiman and Kessler, 2014), in which the salient constituent is the access unit for the whole compound retrieval. The strategies applied by L2 Chinese learners can thus be illustrated with this model hinging with word segmentation mechanism in Chinese. The L2 Chinese learners processed a span of characters in parallel and picked the salient characters for lexicon mapping. The activation of words depended on their language using experience, contexts and perhaps lifestyle. This model can be applied to explain the misrecognition as well. The participants picked the salient characters 步 “step” and 游 “swim/travel” in their respective expressions but activated the wrong words composed by these characters though seeming contextually appropriate.

The interactive model of decoding and linguistic comprehension facilitated L2 Chinese learners’ reading comprehension of split SCs. When the character decoding process failed, linguistic comprehension (structure analysis) took the charge. We are not proposing the bottom-up reading model, in which decoding precedes linguistic comprehension. But as Gough (1972) commented, guessing is helpful but not a sign of normal reading. It is a result of poor decoding (Nicholson, 1993). Thus, the interactive model applied by L2 Chinese learners was double-edged. While assisting L2 Chinese learners to overcome decoding breakdowns, it may block the access to gestalt lexical semantics of SCs and therefore lead to unsuccessful reading comprehension. Unlike native speakers who also apply the interactive model (e.g., Yang, 2021), L2 Chinese learners do not have sufficient vocabulary knowledge, morphological awareness, and other relevant knowledge to guarantee successful guessing. When the insertions in split SCs were modifiers and nominal classifiers, the saliency of the tail character was promoted. In the reading comprehension of such structures, the syntactic structure analysis may overwhelm the necessary morphological analysis and encourages L2 Chinese learners to comprehend the character independently from SCs. Therefore, the learners may fail to grasp the accurate gestalt lexical semantics of SCs.

For the third research question, we assumed that as Spanish following PLI does not allow syntactic analysis to compounds, the native linguistic knowledge may actively block the morphological analysis and lead the L2 Chinese readers to process the split SCs with syntactic strategies. However, it was only partially correct, as L2 Chinese participants only struggled with split SCs of the salient V-O structure and were not sensitive to the unsalient V-O structure in general. They also ignored the insertions in some cases. For example, 4 participants neglected the approximate quantifier 点 “a bit” in 学点习 “study a bit” in their interpretations, and none of the 27 participants showed or expressed any confusion with SCs separated by aspect markers. These insertions in split SCs providing aspectual or other action related information are supposed to be comprehended with verbal constituents (either the first composing morpheme or the whole SCs) without salient cues for objects. Thus, L2 Chinese learners only needed to access the verbal semantics without analyzing the internal compound structure. In contrast, when the V-O structure of split SCs was promoted, the L2 Chinese learners, in lack of compound awareness, may be confused when handling gestalt lexical semantics and phrasal structures at the same time. In other words, PLI held in their L1 morphological knowledge obstructs their whole-word recognition of SCs in salient V-O structures. The recognition accuracy rate of S2 and S12 in Table 5 approved such an assumption. 饭 in 吃好多好多饭 (S2) was overtly expressed as *arroz* “rice” by 2 participants, but such a case was not found in S12. The insertion in S2 was a quantitative expression, and in S12 was the aspect marker 着. The former indicated the quantity of food, but the food was not necessarily to be rice. The awareness of the V-O structure boosted by the insertion misled the participants’ comprehension of the tail character. In addition, when doing the second task, most participants stated that it was more difficult to figure out the syntactic category of non-split SCs than to interpret the sentences involving their split forms. They had no problem with recognizing and translating these non-split forms, but the conscious awareness of the V-O structure of some SCs contradicts their existing L1 morphological knowledge.

In sum, the split presentation influences the SCs’ recognition among L2 Chinese learners in the reading comprehension. The successful recognition of split SCs requires an interactive model of both decoding and linguistic comprehension. What makes this task more challenging for L2 Chinese learners is their L1 linguistic knowledge that actively blocks the morphological analysis in reading split SCs, especially when the V-O relation between the two composing morphemes is salient.

## Conclusion

This study probed into L2 Chinese learners’ in-text word reading comprehension, with a particular focus on the reading process of Chinese SCs in the split forms. The results suggested that L2 Chinese learners found processing the split SCs challenging, especially when the split forms were in salient V-O structures. The morphological knowledge held in the L2 Chinese learns’ native language was not an obvious obstacle for reading split SCs in the unsalient V-O structures, but the L1 knowledge actively blocked morphological analysis in reading split SCs in the salient V-O structures. The findings indicated that in the reading comprehension of complex Chinese words, both compound awareness and syntactic structure awareness could be activated, but the awareness of syntactic structure might obstruct, to some degree, the L2 Chinese reading comprehension.

This study underscores the importance of appropriate linguistic comprehension in Chinese word reading comprehension and suggested the possibility of competence between the awareness of morphological structures and syntactic structures in L2 Chinese word reading comprehension. Such competence, as discussed above, was induced by the L2 learners’ L1 knowledge. The challenges faced by L2 Chinese learners in the reading comprehension of split SCs were due to the conflicts between the gestalt lexical semantics of the compounds and the salient syntactic relation between the composing characters. The increase of Chinese proficiency did not seem to efficiently resolve the conflicts or ease the competition.

This study also added evidence to the existing body of literature about the influence of the character organization on word reading comprehension. The empirical data suggested that similar to the transposed-word effect, in L2 Chinese reading comprehension, the discontinuous presentation of composing characters was able to activate not only the representation of the compound itself but also words of similar forms. The L2 Chinese readers were observed to apply the interactive model of linguistic analysis and word/character decoding in reading comprehension, and the model facilitated L2 Chinese learners to overcome the failure of word decoding. However, it should be addressed that this interactive model may also lead to unsuccessful reading comprehension for L2 learners of low morphological awareness as it could obstruct the learners’ access to gestalt lexical semantics.

The results also shed some light on the conundrum about the basic processing units in less advanced Chinese reading comprehension. The L2 Chinese readers tended to treat the composing characters as separate single-character words when the salient V-O relation between the characters was recognized, but they did not hesitate to decode the SCs of the unsalient V-O structure as holistic forms despite being presented in split forms. These observations suggest that at least for L2 Chinese readers, the basic processing units are words (including the units they identified as words, such as 泳).

Also, we would hasten to point out that we did not claim that L2 learners’ Chinese language proficiency and participants familiarity with the compounds were of no relation to the recognizing of split SCs. The size of participants was not large, and these factors did not show significant influence in the current study. Also, the participants’ previous HSK/other Chinese language test results were used as the reference for their Chinese proficiency levels, but these results may not correctly reflect their Chinese proficiency at the time of testing. Despite this, the current study allowed us to focus on the structure of the expressions and identity many details in L2 Chinese learners’ reading process. Thus, future studies can investigate the developmental differences in the reading of split SCs of various structures and productivity.

## Data Availability Statement

The raw data supporting the conclusions of this article will be made available by the authors, without undue reservation.

## Ethics Statement

The studies involving human participants were reviewed and approved by School of international studies, Zhejiang University. The patients/participants provided their written informed consent to participate in this study.

## Author Contributions

ZL and YW conceived and designed this study. YD collected the data under the guidance of ZL and YW. ZL and YD conducted the data analysis and interpreted the results. ZL drafted the manuscript. All authors revised and approved the final manuscript.

## Author Disclaimer

All views expressed are those of the authors and not of the sponsoring organizations.

## Conflict of Interest

The authors declare that the research was conducted in the absence of any commercial or financial relationships that could be construed as a potential conflict of interest.

## Publisher’s Note

All claims expressed in this article are solely those of the authors and do not necessarily represent those of their affiliated organizations, or those of the publisher, the editors and the reviewers. Any product that may be evaluated in this article, or claim that may be made by its manufacturer, is not guaranteed or endorsed by the publisher.
