# Supplementary material for: Spanish L2 Chinese Learners’ Awareness of Morpho-Syntactic Structures in the Reading Comprehension of Splittable Compounds
Source: Front Psychol. 2022 Jan 7;12:783869. doi: 10.3389/fpsyg.2021.783869 (PMC8777270; doi:10.3389/fpsyg.2021.783869)
Supplement: Supplementary file 1 [file Table_1.docx]

Table 1. *Separability of 8 selected SCs*

| **SC** | **说话**  to talk | **吃饭**  to eat | **跳舞**  to dance | **拍照**  to take photos | **排队**  to queue | **跑步**  to jog | **游泳**  to swim | **学习**  study |
| --- | --- | --- | --- | --- | --- | --- | --- | --- |
| **Separability** | 0.408 | 0.215 | 0.191 | 0.187 | 0.153 | 0.088 | 0.011 | 0.001 |

Table 2. *Mean accuracies (SDs) of interpreting responses*

|  | **Sentences** | **Numbers of Items** | **Mean** | **SDs** | **Min** | **Max** |
| --- | --- | --- | --- | --- | --- | --- |
| S1 | 公交车来了，他快速跑了几步。 | 25 | 3.32 | 2.361694307 | 0 | 6 |
|  | The bus was coming, and he quickly ran a few steps |  |  |  |  |  |
| S2 | 我要吃好多好多饭。 | 25 | 5.44 | 0.941488184 | 3 | 6 |
|  | I want to eat lots and lots of food. |  |  |  |  |  |
| S3 | 学生在食堂排了很长的队。 | 25 | 5.2 | 1.959591794 | 4 | 6 |
|  | The students formed a long queue in the food hall. |  |  |  |  |  |
| S4 | 我们和老师昨天照了很多相。 | 25 | 5.76 | 1.175755077 | 0 | 6 |
|  | The professor and us took a lot of pictures yesterday |  |  |  |  |  |
| S5 | 今天他还会跳两支舞。 | 25 | 5.04 | 1.928315327 | 0 | 6 |
|  | Today, he will still dance two dances |  |  |  |  |  |
| S6 | 他只能游三十米的泳。 | 25 | 5.36 | 1.382172203 | 1 | 6 |
|  | He can only swim 30 metres |  |  |  |  |  |
| S7 | 明天考试，今天我要学点习。 | 25 | 5.6 | 0.8 | 4 | 6 |
|  | There is an exam tomorrow, so I need to study a bit |  |  |  |  |  |
| S8 | 他每天晚上跑三十分钟步。 | 25 | 5.8 | 0.979795897 | 1 | 6 |
|  | He jogs 30 minutes every evening |  |  |  |  |  |
| S9 | 他排了一晚上的队，所以很累。 | 25 | 4.76 | 2.232129029 | 0 | 6 |
|  | He queued the whole night so was very tired |  |  |  |  |  |
| S10 | 在party上，大家都跳了舞。 | 25 | 6 | 0 | 6 | 6 |
|  | At the party, everyone danced |  |  |  |  |  |
| S11 | 他每周游两次泳。 | 25 | 5.72 | 1.00079968 | 1 | 6 |
|  | He swims twice ever week |  |  |  |  |  |
| S12 | 他过去两年都没有学过习。 | 25 | 5.84 | 0.611882342 | 3 | 6 |
|  | He hasn't been studying for the past two years |  |  |  |  |  |
| S13 | 他现在正吃着饭。 | 25 | 5.88 | 0.324961536 | 5 | 6 |
|  | He is eating right now |  |  |  |  |  |
| S14 | 今天我们先出去游个泳吧。 | 25 | 5.52 | 1.36 | 1 | 6 |
|  | Let's go swimming first today |  |  |  |  |  |
| S15 | 他早上想去海边跑个步。 | 25 | 5.16 | 1.826033954 | 1 | 6 |
|  | He wants to jog at the sea. |  |  |  |  |  |
| S16 | 他在外面跑了一会儿步。 | 11 | 4.454545455 | 2.189380833 | 1 | 6 |
|  | He jogged outside for a while. |  |  |  |  |  |
| S17 | 老师跟他说了几句话。 | 6 | 5.333333333 | 0.942809042 | 4 | 6 |
|  | The teacher spoke a few words with him. |  |  |  |  |  |
| S18 | 晚上我们和老师一起吃了很久的饭。 | 1 | 4 | 0 | 4 | 4 |
|  | The proferssor and us had a long meal at night. |  |  |  |  |  |
|  | Total |  | 5.232659933 | 0.689245972 |  |  |

Table 3 *Mean accuracies (SDs) for all measures of each SC*

|  | **SC** | **Numbers of Items** | **Familiarity** | | | | | | |  | | **Lexical (0) /phrasal (1) status** | | | | |
| --- | --- | --- | --- | --- | --- | --- | --- | --- | --- | --- | --- | --- | --- | --- | --- | --- |
|  |  |  | **Mean** | **SDs** | | **Min** | | | **Max** | |  | | **Mean** | **SDs** | **Min** | **Max** |
| SC1 | 吃饭 | 25 | 4.96 | | 0.192154 | | 4 | 5 | |  | | 0.68 | | 0.444522 | 0 | 1 |
|  | to eat |  |  | |  | |  |  | |  | |  | |  |  |  |
| SC2 | 跑步 | 25 | 4.52 | | 1.04218705 | | 0 | 5 | |  | | 0.38 | | 0.474974 | 0 | 1 |
|  | to run |  |  | |  | |  |  | |  | |  | |  |  |  |
| SC3 | 排队 | 25 | 3.4 | | 1.568929081 | | 0 | 5 | |  | | 0.5625 | | 0.463512 | 0 | 1 |
|  | to queue |  |  | |  | |  |  | |  | |  | |  |  |  |
| SC4 | 拍照 | 25 | 3.98 | | 1.117847634 | | 2 | 5 | |  | | 0.7 | | 0.447214 | 0 | 1 |
|  | to take photos |  |  | |  | |  |  | |  | |  | |  |  |  |
| SC5 | 跳舞 | 25 | 4.64 | | 0.612686392 | | 3 | 5 | |  | | 0.42 | | 0.483322 | 0 | 1 |
|  | to dance |  |  | |  | |  |  | |  | |  | |  |  |  |
| SC6 | 游泳 | 25 | 4.56 | | 0.788377106 | | 2 | 5 | |  | | 0.34 | | 0.463033 | 0 | 1 |
|  | to swim |  |  | |  | |  |  | |  | |  | |  |  |  |
| SC7 | 说话 | 25 | 4.68 | | 0.663324958 | | 3 | 5 | |  | | 0.62 | | 0.474974 | 0 | 1 |
|  | to talk |  |  | |  | |  |  | |  | |  | |  |  |  |
| SC8 | 学习 | 25 | 4.96 | | 0.192153785 | | 4 | 5 | |  | | 0.2 | | 0.4 | 0 | 1 |
|  | to study |  |  | |  | |  |  | |  | |  | |  |  |  |
|  | Total |  | 4.46 | | 0.493603431 | |  |  | |  | | 0.49 | | 0.1680469 |  |  |

Table 4 *Pearson Correlation analysis with SC familiarity and accuracy scores*

| **a.** the familiarity of 吃饭 and the score of translation | | | |
| --- | --- | --- | --- |
|  | F_吃饭 | S2 | S13 |
| F_吃饭 | 1 |  |  |
| S2 | 0.529^*^ | 1 |  |
| S13 | -0.075 | -0.089 | 1 |
| *N=25; significance level: ^*^ p<0.01 (2-tailed); F_吃饭 is the familiarity of 吃饭; S2 is the score of sentence-2 translation; S13 is the score of sentence-13 translation.* | | | |

| **b.** the familiarity of 跑步 and the score of translation | | | | | |  |
| --- | --- | --- | --- | --- | --- | --- |
|  | F_跑步 | S1 | S8 | S15 | S16 | |
| F_跑步 | 1 |  |  |  |  | |
| S1 | 0.109 | 1 |  |  |  | |
| S8 | -0.092 | 0.201 | 1 |  |  | |
| S15 | 0.328 | 0.201 | 0.465^*^ | 1 |  | |
| S16 | 0.390 | 0.088 | 0.499 | 0.966^*^ | 1 | |
| *N=25; significance level: ^*^ p<0.01 (2-tailed); F_跑步 is the familiarity of 跑步; S1 is the score of sentence-1 translation; S8 is the score of sentence-8 translation; S15 is the score of sentence-15 translation; S16 is the score of sentence-16 translation.* | | | | | |  |

| **c.** the familiarity of 排队 and the score of translation | | | |
| --- | --- | --- | --- |
|  | F_排队 | S3 | S9 |
| F_排队 | 1 |  |  |
| S3 | 0.638^*^ | 1 |  |
| S9 | 0.744^*^ | 0.779^*^ | 1 |
| *N=25; significance level: ^*^ p<0.01 (2-tailed); F_排队is the familiarity of 排队; S3 is the score of sentence-3 translation; S9 is the score of sentence-9 translation.* | | | |

| **d.** the familiarity of 拍照 and the score of translation | | |
| --- | --- | --- |
|  | F_拍照 | S4_camara |
| F_拍照 | 1 |  |
| S4 | 0.572^*^ | 1 |
| *N=25; significance level: ^*^ p<0.01 (2-tailed); F_拍照is the familiarity of 拍照; S4 is the score of sentence-4 translation.* | | |

| **e.** the familiarity of 跳舞 and the score of translation | | |
| --- | --- | --- |
|  | F_跳舞 | S5 |
| F_跳舞 | 1 |  |
| S5 | -0.021 | 1 |
| *N=25; F_跳舞is the familiarity of 跳舞; S5 is the score of sentence-5 translation.* | | |

| **f.** the familiarity of 游泳 and the score of translation | | | | |
| --- | --- | --- | --- | --- |
|  | F_游泳 | S6 | S11 | S14 |
| F_游泳 | 1 |  |  |  |
| S6 | 0.646^*^ | 1 |  |  |
| S11 | 0.095 | -0.13 | 1 |  |
| S14 | -0.01 | -0.121 | 0.636^*^ | 1 |
| *N=25; significance level: ^*^ p<0.01 (2-tailed); F_游泳 is the familiarity of 游泳; S6 is the score of sentence-6 translation; S11 is the score of sentence-11 translation; S14 is the score of sentence-14 translation.* | | | | |

| **g.** the familiarity of 说话 and the score of translation | | | | | |
| --- | --- | --- | --- | --- | --- |
|  | | F_说话 | | S17_speak | |
| F_说话 | | 1 | |  | |
| S17 | | 0.926^*^ | | 1 | |
| *N=25; significance level: ^*^ p<0.01 (2-tailed); F_说话 is the familiarity of说话; S17 is the score of sentence-17 translation.* | | | | | |
| **h.** the familiarity of 学习 and the score of translation | | | | | |
|  | F_study | | S7_study | | S12_study |
| F_study | 1 | |  | |  |
| S7 | -0.102 | | 1 | |  |
| S12 | -0.053 | | 0.36 | | 1 |
| *N=25; F_study is the familiarity of学习; S7 is the score of sentence-7 translation; S12 is the score of sentence-12 translation.* | | | | | |

Table 5. *Multiple comparisons between insertion types*

| **a.** the familiarity of 吃饭 and the score of translation | | | |
| --- | --- | --- | --- |
|  | F_吃饭 | S2 | S13 |
| F_吃饭 | 1 |  |  |
| S2 | 0.529^*^ | 1 |  |
| S13 | -0.075 | -0.089 | 1 |
| *N=25; significance level: ^*^ p<0.01 (2-tailed); F_吃饭 is the familiarity of 吃饭; S2 is the score of sentence-2 translation; S13 is the score of sentence-13 translation.* | | | |

| **b.** the familiarity of 跑步 and the score of translation | | | | | |  |
| --- | --- | --- | --- | --- | --- | --- |
|  | F_跑步 | S1 | S8 | S15 | S16 | |
| F_跑步 | 1 |  |  |  |  | |
| S1 | 0.109 | 1 |  |  |  | |
| S8 | -0.092 | 0.201 | 1 |  |  | |
| S15 | 0.328 | 0.201 | 0.465^*^ | 1 |  | |
| S16 | 0.390 | 0.088 | 0.499 | 0.966^*^ | 1 | |
| *N=25; significance level: ^*^ p<0.01 (2-tailed); F_跑步 is the familiarity of 跑步; S1 is the score of sentence-1 translation; S8 is the score of sentence-8 translation; S15 is the score of sentence-15 translation; S16 is the score of sentence-16 translation.* | | | | | |  |

| **c.** the familiarity of 排队 and the score of translation | | | |
| --- | --- | --- | --- |
|  | F_排队 | S3 | S9 |
| F_排队 | 1 |  |  |
| S3 | 0.638^*^ | 1 |  |
| S9 | 0.744^*^ | 0.779^*^ | 1 |
| *N=25; significance level: ^*^ p<0.01 (2-tailed); F_排队is the familiarity of 排队; S3 is the score of sentence-3 translation; S9 is the score of sentence-9 translation.* | | | |

| **d.** the familiarity of 拍照 and the score of translation | | |
| --- | --- | --- |
|  | F_拍照 | S4_camara |
| F_拍照 | 1 |  |
| S4 | 0.572^*^ | 1 |
| *N=25; significance level: ^*^ p<0.01 (2-tailed); F_拍照is the familiarity of 拍照; S4 is the score of sentence-4 translation.* | | |

| **e.** the familiarity of 跳舞 and the score of translation | | |
| --- | --- | --- |
|  | F_跳舞 | S5 |
| F_跳舞 | 1 |  |
| S5 | -0.021 | 1 |
| *N=25; F_跳舞is the familiarity of 跳舞; S5 is the score of sentence-5 translation.* | | |

| **f.** the familiarity of 游泳 and the score of translation | | | | |
| --- | --- | --- | --- | --- |
|  | F_游泳 | S6 | S11 | S14 |
| F_游泳 | 1 |  |  |  |
| S6 | 0.646^*^ | 1 |  |  |
| S11 | 0.095 | -0.13 | 1 |  |
| S14 | -0.01 | -0.121 | 0.636^*^ | 1 |
| *N=25; significance level: ^*^ p<0.01 (2-tailed); F_游泳 is the familiarity of 游泳; S6 is the score of sentence-6 translation; S11 is the score of sentence-11 translation; S14 is the score of sentence-14 translation.* | | | | |

| **g.** the familiarity of 说话 and the score of translation | | | | | |
| --- | --- | --- | --- | --- | --- |
|  | | F_说话 | | S17_speak | |
| F_说话 | | 1 | |  | |
| S17 | | 0.926^*^ | | 1 | |
| *N=25; significance level: ^*^ p<0.01 (2-tailed); F_说话 is the familiarity of说话; S17 is the score of sentence-17 translation.* | | | | | |
| **h.** the familiarity of 学习 and the score of translation | | | | | |
|  | F_study | | S7_study | | S12_study |
| F_study | 1 | |  | |  |
| S7 | -0.102 | | 1 | |  |
| S12 | -0.053 | | 0.36 | | 1 |
| *N=25; F_study is the familiarity of学习; S7 is the score of sentence-7 translation; S12 is the score of sentence-12 translation.* | | | | | |
